# 4,4′-Di-*tert*-butyl-2,2′-[(3a*RS*,7a*RS*)-2,3,3a,4,5,6,7,7a-octa­hydro-1*H*-1,3-benzimidazole-1,3-di­yl)bis­(methyl­ene)]diphenol

**DOI:** 10.1107/S1600536811039171

**Published:** 2011-10-12

**Authors:** Augusto Rivera, Dency José Pacheco, Jaime Ríos-Motta, Michaela Pojarová, Michal Dušek

**Affiliations:** a Departamento de Química, Universidad Nacional de Colombia, Ciudad Universitaria, Bogotá, Colombia; b Institute of Physics, AS CR, v.v.i., Na Slovance 2, 182 21 Praha 8, Czech Republic

## Abstract

In the title compound, C_29_H_42_N_2_O_2_, the heterocyclic ring has a twist conformation. The cyclohexane ring adopts a chair conformation. The dihedral angle between the aromatic rings is 32.74 (6)°. The mol­ecular conformation is stabilized by two intramolecular O—H⋯N hydrogen bonds with graph-set motif *S*(6). The crystal packing is stabilized by C—H⋯O and C—H⋯π inter­actions.

## Related literature

For related structures, see: Rivera *et al.* (2009 ▶, 2010 ▶). For puckering parameters, see: Cremer & Pople (1975 ▶). For hydrogen-bond graph-set nomenclature, see: Bernstein *et al.* (1995 ▶).
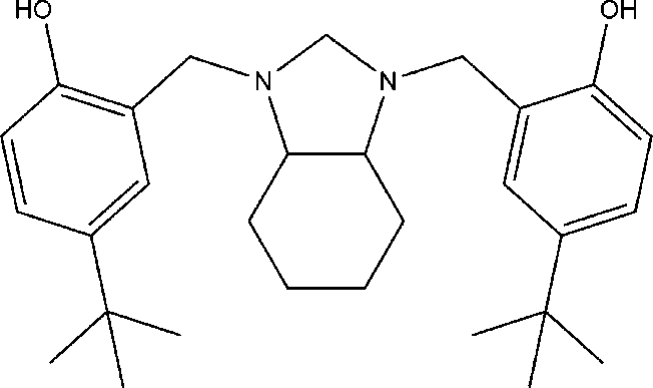


## Experimental

### 

#### Crystal data

C_29_H_42_N_2_O_2_
*M* *_r_* = 450.65
Triclinic, 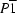

*a* = 6.2383 (2) Å
*b* = 14.2296 (5) Å
*c* = 15.6530 (6) Å
α = 105.942 (3)°
β = 95.737 (3)°
γ = 98.041 (3)°
*V* = 1308.87 (8) Å^3^
*Z* = 2
Cu *K*α radiation
μ = 0.55 mm^−1^
*T* = 120 K
0.22 × 0.10 × 0.08 mm

#### Data collection

Agilent Xcalibur Atlas Gemini Ultra diffractometer
Absorption correction: analytical (*CrysAlis PRO*; Agilent, 2011) ▶ *T*_min_ = 0.246, *T*_max_ = 0.581
28373 measured reflections
4676 independent reflections
3632 reflections with *I* > 2σ(*I*)
*R*_int_ = 0.139

#### Refinement

*R*[*F* ^2^ > 2σ(*F* ^2^)] = 0.054
*wR*(*F* ^2^) = 0.143
*S* = 1.01
4676 reflections
304 parameters
2 restraints
H-atom parameters constrained
Δρ_max_ = 0.21 e Å^−3^
Δρ_min_ = −0.27 e Å^−3^

### 

Data collection: *CrysAlis PRO* (Agilent, 2011 ▶); cell refinement: *CrysAlis PRO*; data reduction: *CrysAlis PRO*; program(s) used to solve structure: *SHELXS97* (Sheldrick, 2008 ▶); program(s) used to refine structure: *SHELXL97* (Sheldrick, 2008 ▶); molecular graphics: *DIAMOND* (Brandenburg & Putz, 2005 ▶); software used to prepare material for publication: *publCIF* (Westrip, 2010 ▶).

## Supplementary Material

Crystal structure: contains datablock(s) I, global. DOI: 10.1107/S1600536811039171/bt5652sup1.cif

Structure factors: contains datablock(s) I. DOI: 10.1107/S1600536811039171/bt5652Isup2.hkl

Additional supplementary materials:  crystallographic information; 3D view; checkCIF report

## Figures and Tables

**Table 1:** Hydrogen-bond geometry (Å, °) *Cg*3 and *Cg*4 are the centroids of the C9–C14 and C20–C25 benzene rings, respectively.

*D*—H⋯*A*	*D*—H	H⋯*A*	*D*⋯*A*	*D*—H⋯*A*
O1—H1*O*1⋯N1	0.98	1.77	2.6585 (18)	148
O2—H1*O*2⋯N2	0.89	1.86	2.6794 (18)	153
C2—H2⋯O2^i^	0.98	2.45	3.367 (18)	155
C5—H5*B*⋯*Cg*3^ii^	0.96	2.87	3.625 (2)	135
C19—H19*A*⋯*Cg*4^iii^	0.96	2.84	3.6722 (18)	144

Symmetry codes: (i) 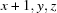; (ii) 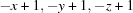; (iii) 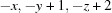.

**Fig. 1.**
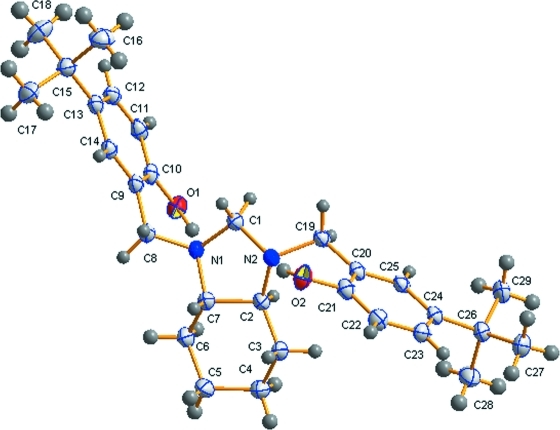
A view of (I) with the numbering scheme. Displacement ellipsoids are drawn at the 50% probability level.

**Fig. 2.**
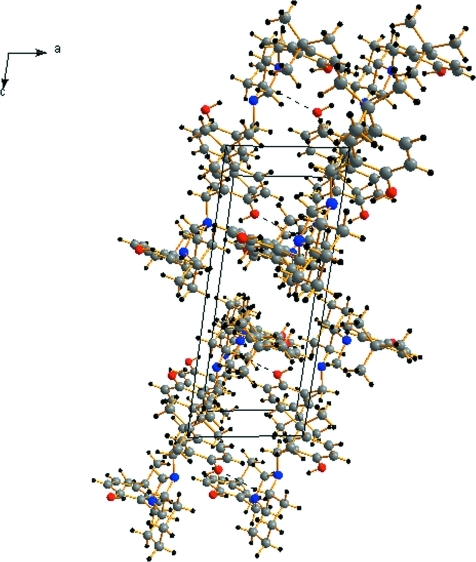
Packing of the molecules of the title compound view along b axis.

## supplementary crystallographic information

### Comment

It is known that intramolecular O—H···N hydrogen bonds in Mannich and Schiff bases play a key role in the themodynamic stability of these bases. In our group, research has been focused on the development of novel *di*-Mannich bases (Rivera *et al.*, 2009, 2010) and their hydrogen-bonded structures. In this work, we report the crystal structure of 4,4'-ditertbutyl-3,3',5,5'-tetramethyl-2,2'-[(3aR,7aR/3aS,7aS)-2,3,3a,4,5,6,7, 7a-octahydro-1*H*-1,3-benzimidazole-1,3-diyl)bis(methylene)]diphenol (I) as hydrogen bonding model. The molecular structure and atom-numbering scheme for (I) are shown in Fig.1. The bond lengths are normal and comparable to the corresponding values observed in the related structures (Rivera *et al.*, 2009, 2010). The aromatic rings (C9—C14; C20—C25) are essentially planar with the maximum deviation from planarity being 0.0094 (19)° for atom C18.

As with related structures in this series, the heterocyclic ring has a twisted conformation on C2—C7, (Q(2)= 0.4380 (10) Å, φ = 120.3 (2)°, (Cremer & Pople, 1975), and as is typical for such Mannich bases and the molecular conformation is stabilized by two intramolecular O—H···N hydrogen-bond interaction with set graph motif S(6) (Bernstein *et al.* 1995). However, contrary to other structures, where the difference in the hydrogen bond lengths may be considered to be negligible, the two observed intramolecular hydrogen bond distances were different (Table 1). Considering the similarity of the chemical environment around of both nitrogen atoms, it is then surprising to see the difference in the O—H distances between O2—H2 [O—H = 0.89 Å (2)] and O1—H1 [O—H = 0.98 Å (18)], which is longer compared to the previous compounds (Rivera *et al.*, 2009, 2010). Our observation for this difference can be correlated to the difference in the participation of each one of oxygen atoms in hydrogen-bond networks. Although a hydroxyl group is involved as an acceptor hydrogen bond in an intermolecular hydrogen bond, the other is a non-intermolecular-hydrogen-bonded hydroxyl group. The intermolecular hydrogen bonds [C2—H2A···O2*^i^*, symmetry code: *x* + 1, *y*, *z*] bridge the molecules through *head-to-tail* into a one-dimensional chain running parallel to the *a* axis (Figure 2). These chains are stabilized by C—H···π interactions (Table 1).

### Experimental

To a dioxane:water (7 ml) solution of the aminal (2*R*,7*R*,11*S*,16S)-1,8,10,17- tetraazapentacyclo[8.8.1.1^8,17^.0^2,7^.0^11,16^]- icosane (276 mg, 1.00 mmol) was added dropwise a dioxane solution (3 ml) containing two equivalents of *p*-tertbutylphenol (300 mg, 2.00 mmol). The mixture was refluxed for about 10 h. The solvent was evaporated under reduced pressure until a sticky residue appeared. The product was purified by chromatography on a silica column, and subjected to gradient elution with benzene:ethyl acetate (yield 47%, *M*.p. = 430–431 K). Single crystals of racemic (I) were grown from a chloroform: methanol solution by slow evaporation of the solvent at room temperature over a period of about 2 weeks.

### Refinement

According to common practice H atoms bonded C atoms were kept in ideal positions with C—H distance 0.96 Å during the refinement. The isotropic displacement parameters of the hydrogen atoms were calculated as 1.2**U*_eq_ of the parent atom. The distance between hydrogen and oxygen atom in hydroxyl group was fixed to the distance 0.87 Å. The quality of the crystals was very low. The selected crystal for measurement was the best one from several attempts.

### Crystal data

**Table:**

C_29_H_42_N_2_O_2_	*Z* = 2
*M**_r_* = 450.65	*F*(000) = 492
Triclinic, *P*1	*D*_x_ = 1.143 Mg m^−^^3^
Hall symbol: -P 1	Cu *K*α radiation, λ = 1.5418 Å
*a* = 6.2383 (2) Å	Cell parameters from 9605 reflections
*b* = 14.2296 (5) Å	θ = 3.0–67.2°
*c* = 15.6530 (6) Å	µ = 0.55 mm^−^^1^
α = 105.942 (3)°	*T* = 120 K
β = 95.737 (3)°	Prism, colourless
γ = 98.041 (3)°	0.22 × 0.10 × 0.08 mm
*V* = 1308.87 (8) Å^3^	

### Data collection

**Table:**

Agilent Xcalibur Atlas Gemini Ultra diffractometer	4676 independent reflections
Radiation source: Enhance Ultra (Cu) X-ray Source	3632 reflections with *I* > 2σ(*I*)
mirror	*R*_int_ = 0.139
Detector resolution: 10.3784 pixels mm^-1^	θ_max_ = 67.3°, θ_min_ = 3.0°
Rotation method data acquisition using ω scans	*h* = −7→7
Absorption correction: analytical (*CrysAlis PRO*; Agilent, 2011)	*k* = −17→17
*T*_min_ = 0.246, *T*_max_ = 0.581	*l* = −18→18
28373 measured reflections	

### Refinement

**Table:**

Refinement on *F*^2^	Primary atom site location: structure-invariant direct methods
Least-squares matrix: full	Secondary atom site location: difference Fourier map
*R*[*F*^2^ > 2σ(*F*^2^)] = 0.054	Hydrogen site location: inferred from neighbouring sites
*wR*(*F*^2^) = 0.143	H-atom parameters constrained
*S* = 1.01	*w* = 1/[σ^2^(*F*_o_^2^) + (0.0802*P*)^2^] where *P* = (*F*_o_^2^ + 2*F*_c_^2^)/3
4676 reflections	(Δ/σ)_max_ < 0.001
304 parameters	Δρ_max_ = 0.21 e Å^−^^3^
2 restraints	Δρ_min_ = −0.27 e Å^−^^3^

### Special details

**Table:**

Geometry. All e.s.d.'s (except the e.s.d. in the dihedral angle between two l.s. planes) are estimated using the full covariance matrix. The cell e.s.d.'s are taken into account individually in the estimation of e.s.d.'s in distances, angles and torsion angles; correlations between e.s.d.'s in cell parameters are only used when they are defined by crystal symmetry. An approximate (isotropic) treatment of cell e.s.d.'s is used for estimating e.s.d.'s involving l.s. planes.
Refinement. Refinement of *F*^2^ against ALL reflections. The weighted *R*-factor *wR* and goodness of fit *S* are based on *F*^2^, conventional *R*-factors *R* are based on *F*, with *F* set to zero for negative *F*^2^. The threshold expression of *F*^2^ > σ(*F*^2^) is used only for calculating *R*-factors(gt) *etc*. and is not relevant to the choice of reflections for refinement. *R*-factors based on *F*^2^ are statistically about twice as large as those based on *F*, and *R*- factors based on ALL data will be even larger. The H atoms were all located in a difference map, but those attached to carbon atoms were repositioned geometrically. The isotropic temperature parameters of hydrogen atoms were calculated as 1.2**U*_eq_ of the parent atom. The distance between hydrogen and oxygen atom in hydroxyl group was fixed to the distance 0.87 Å.

### Fractional atomic coordinates and isotropic or equivalent isotropic displacement parameters (Å^2^)

**Table:**

	*x*	*y*	*z*	*U*_iso_*/*U*_eq_	
O1	0.7423 (2)	0.60526 (9)	0.68014 (9)	0.0334 (3)	
H1O1	0.6083	0.5598	0.6803	0.040*	
O2	−0.2846 (2)	0.45402 (10)	0.80993 (9)	0.0357 (3)	
H1O2	−0.1676	0.4721	0.7865	0.043*	
N1	0.3185 (2)	0.53749 (10)	0.67034 (9)	0.0253 (3)	
N2	0.1289 (2)	0.48505 (10)	0.77727 (9)	0.0244 (3)	
C1	0.2544 (3)	0.57266 (12)	0.76055 (11)	0.0266 (4)	
H1A	0.3831	0.6001	0.8055	0.032*	
H1B	0.1650	0.6235	0.7624	0.032*	
C2	0.1894 (3)	0.39934 (12)	0.71235 (11)	0.0253 (4)	
H2	0.3379	0.3924	0.7336	0.030*	
C3	0.0424 (3)	0.29864 (12)	0.68838 (11)	0.0306 (4)	
H3A	−0.1059	0.3035	0.6670	0.037*	
H3B	0.0398	0.2748	0.7407	0.037*	
C4	0.1353 (4)	0.22741 (14)	0.61467 (13)	0.0379 (4)	
H4A	0.2772	0.2182	0.6392	0.045*	
H4B	0.0393	0.1633	0.5958	0.045*	
C5	0.1597 (4)	0.26467 (14)	0.53294 (13)	0.0403 (5)	
H5A	0.0157	0.2649	0.5033	0.048*	
H5B	0.2295	0.2195	0.4908	0.048*	
C6	0.2954 (3)	0.36938 (14)	0.55839 (12)	0.0363 (4)	
H6A	0.4458	0.3683	0.5803	0.044*	
H6B	0.2942	0.3937	0.5062	0.044*	
C7	0.1961 (3)	0.43638 (12)	0.63083 (11)	0.0280 (4)	
H7	0.0469	0.4394	0.6068	0.034*	
C8	0.2926 (3)	0.60451 (12)	0.61501 (11)	0.0276 (4)	
H8A	0.1475	0.6216	0.6156	0.033*	
H8B	0.3069	0.5710	0.5534	0.033*	
C9	0.4626 (3)	0.69832 (12)	0.64956 (11)	0.0255 (4)	
C10	0.6796 (3)	0.69352 (12)	0.67912 (11)	0.0263 (4)	
C11	0.8342 (3)	0.77961 (13)	0.70752 (11)	0.0299 (4)	
H11	0.9777	0.7768	0.7279	0.036*	
C12	0.7777 (3)	0.87005 (13)	0.70595 (11)	0.0290 (4)	
H12	0.8842	0.9270	0.7254	0.035*	
C13	0.5642 (3)	0.87735 (12)	0.67573 (11)	0.0268 (4)	
C14	0.4105 (3)	0.78981 (12)	0.64918 (11)	0.0263 (4)	
H14	0.2662	0.7930	0.6303	0.032*	
C15	0.4973 (3)	0.97661 (13)	0.67471 (12)	0.0323 (4)	
C16	0.4002 (4)	1.01787 (15)	0.76044 (15)	0.0449 (5)	
H16A	0.2771	0.9708	0.7636	0.058*	
H16B	0.3534	1.0791	0.7597	0.058*	
H16C	0.5090	1.0295	0.8118	0.058*	
C17	0.3244 (4)	0.96239 (14)	0.59319 (14)	0.0426 (5)	
H17A	0.1925	0.9222	0.5988	0.055*	
H17B	0.3783	0.9302	0.5393	0.055*	
H17C	0.2942	1.0259	0.5905	0.055*	
C18	0.6922 (4)	1.05243 (15)	0.67086 (17)	0.0472 (5)	
H18A	0.6425	1.1116	0.6653	0.061*	
H18B	0.7620	1.0252	0.6200	0.061*	
H18C	0.7948	1.0682	0.7249	0.061*	
C19	0.1725 (3)	0.48777 (12)	0.87265 (11)	0.0260 (4)	
H19A	0.1612	0.5529	0.9106	0.031*	
H19B	0.3203	0.4765	0.8860	0.031*	
C20	0.0128 (3)	0.41008 (12)	0.89290 (11)	0.0259 (4)	
C21	−0.2101 (3)	0.39674 (13)	0.85995 (11)	0.0288 (4)	
C22	−0.3563 (3)	0.32395 (13)	0.87683 (12)	0.0321 (4)	
H22	−0.5041	0.3147	0.8546	0.039*	
C23	−0.2838 (3)	0.26467 (13)	0.92669 (12)	0.0299 (4)	
H23	−0.3847	0.2161	0.9374	0.036*	
C24	−0.0639 (3)	0.27594 (12)	0.96120 (11)	0.0269 (4)	
C25	0.0807 (3)	0.34982 (12)	0.94307 (10)	0.0256 (4)	
H25	0.2283	0.3592	0.9655	0.031*	
C26	0.0192 (3)	0.20602 (13)	1.01118 (11)	0.0304 (4)	
C27	−0.1570 (3)	0.16459 (15)	1.05918 (13)	0.0388 (4)	
H27A	−0.2092	0.2185	1.0983	0.050*	
H27B	−0.0958	0.1260	1.0938	0.050*	
H27C	−0.2763	0.1233	1.0155	0.050*	
C28	0.0852 (4)	0.11927 (15)	0.94238 (13)	0.0417 (5)	
H28A	0.1450	0.0767	0.9729	0.054*	
H28B	0.1930	0.1446	0.9109	0.054*	
H28C	−0.0413	0.0823	0.9004	0.054*	
C29	0.2192 (3)	0.25932 (15)	1.08137 (12)	0.0367 (4)	
H29A	0.1840	0.3173	1.1218	0.048*	
H29B	0.3383	0.2785	1.0518	0.048*	
H29C	0.2609	0.2155	1.1145	0.048*	

### Atomic displacement parameters (Å^2^)

**Table:**

	*U*^11^	*U*^22^	*U*^33^	*U*^12^	*U*^13^	*U*^23^
O1	0.0310 (6)	0.0344 (6)	0.0392 (7)	0.0074 (5)	0.0080 (5)	0.0162 (5)
O2	0.0280 (6)	0.0433 (7)	0.0428 (7)	0.0066 (5)	0.0077 (5)	0.0232 (6)
N1	0.0319 (7)	0.0244 (7)	0.0210 (7)	0.0028 (6)	0.0063 (5)	0.0091 (5)
N2	0.0295 (7)	0.0236 (7)	0.0192 (7)	0.0032 (5)	0.0046 (5)	0.0053 (5)
C1	0.0319 (8)	0.0250 (8)	0.0217 (8)	0.0031 (7)	0.0061 (7)	0.0050 (6)
C2	0.0320 (8)	0.0243 (8)	0.0193 (8)	0.0048 (7)	0.0053 (6)	0.0052 (6)
C3	0.0390 (9)	0.0276 (8)	0.0239 (8)	−0.0001 (7)	0.0072 (7)	0.0071 (7)
C4	0.0531 (11)	0.0270 (9)	0.0314 (10)	0.0018 (8)	0.0096 (8)	0.0062 (7)
C5	0.0603 (13)	0.0302 (9)	0.0262 (9)	0.0008 (9)	0.0121 (9)	0.0029 (8)
C6	0.0512 (11)	0.0321 (9)	0.0246 (9)	0.0017 (8)	0.0127 (8)	0.0071 (7)
C7	0.0340 (9)	0.0264 (8)	0.0224 (8)	0.0006 (7)	0.0044 (7)	0.0072 (7)
C8	0.0319 (8)	0.0285 (8)	0.0230 (8)	0.0009 (7)	0.0025 (7)	0.0112 (7)
C9	0.0285 (8)	0.0294 (8)	0.0191 (8)	0.0017 (7)	0.0063 (6)	0.0086 (6)
C10	0.0293 (8)	0.0320 (9)	0.0215 (8)	0.0063 (7)	0.0099 (6)	0.0113 (7)
C11	0.0262 (8)	0.0398 (10)	0.0240 (8)	0.0024 (7)	0.0056 (7)	0.0110 (7)
C12	0.0304 (9)	0.0311 (9)	0.0224 (8)	−0.0026 (7)	0.0055 (7)	0.0059 (7)
C13	0.0311 (8)	0.0289 (8)	0.0200 (8)	0.0016 (7)	0.0074 (6)	0.0069 (6)
C14	0.0273 (8)	0.0308 (8)	0.0228 (8)	0.0032 (7)	0.0059 (6)	0.0113 (7)
C15	0.0366 (9)	0.0272 (9)	0.0319 (9)	0.0033 (7)	0.0080 (7)	0.0068 (7)
C16	0.0506 (12)	0.0362 (10)	0.0434 (11)	0.0067 (9)	0.0140 (9)	0.0024 (9)
C17	0.0557 (12)	0.0296 (9)	0.0424 (11)	0.0083 (8)	−0.0004 (9)	0.0126 (8)
C18	0.0463 (11)	0.0354 (10)	0.0626 (14)	0.0005 (9)	0.0120 (10)	0.0208 (10)
C19	0.0285 (8)	0.0285 (8)	0.0193 (8)	0.0031 (6)	0.0047 (6)	0.0047 (6)
C20	0.0308 (8)	0.0280 (8)	0.0179 (7)	0.0050 (7)	0.0072 (6)	0.0039 (6)
C21	0.0299 (9)	0.0331 (9)	0.0246 (8)	0.0082 (7)	0.0072 (7)	0.0081 (7)
C22	0.0273 (8)	0.0365 (9)	0.0331 (9)	0.0044 (7)	0.0074 (7)	0.0106 (8)
C23	0.0312 (9)	0.0297 (8)	0.0278 (9)	0.0002 (7)	0.0100 (7)	0.0075 (7)
C24	0.0348 (9)	0.0275 (8)	0.0173 (7)	0.0040 (7)	0.0078 (7)	0.0043 (6)
C25	0.0285 (8)	0.0300 (8)	0.0169 (7)	0.0032 (7)	0.0056 (6)	0.0045 (6)
C26	0.0394 (9)	0.0300 (9)	0.0222 (8)	0.0043 (7)	0.0071 (7)	0.0083 (7)
C27	0.0463 (11)	0.0394 (10)	0.0343 (10)	0.0032 (8)	0.0105 (8)	0.0170 (8)
C28	0.0588 (13)	0.0361 (10)	0.0316 (10)	0.0154 (9)	0.0094 (9)	0.0077 (8)
C29	0.0451 (11)	0.0405 (10)	0.0261 (9)	0.0045 (8)	0.0028 (8)	0.0147 (8)

### Geometric parameters (Å, °)

**Table:**

O1—C10	1.370 (2)	C13—C15	1.531 (2)
O1—H1O1	0.9834	C14—H14	0.9300
O2—C21	1.370 (2)	C15—C18	1.526 (3)
O2—H1O2	0.8859	C15—C16	1.530 (3)
N1—C7	1.465 (2)	C15—C17	1.534 (3)
N1—C8	1.468 (2)	C16—H16A	0.9600
N1—C1	1.478 (2)	C16—H16B	0.9600
N2—C1	1.477 (2)	C16—H16C	0.9600
N2—C2	1.479 (2)	C17—H17A	0.9600
N2—C19	1.479 (2)	C17—H17B	0.9600
C1—H1A	0.9700	C17—H17C	0.9600
C1—H1B	0.9700	C18—H18A	0.9600
C2—C7	1.510 (2)	C18—H18B	0.9600
C2—C3	1.518 (2)	C18—H18C	0.9600
C2—H2	0.9800	C19—C20	1.504 (2)
C3—C4	1.533 (3)	C19—H19A	0.9700
C3—H3A	0.9700	C19—H19B	0.9700
C3—H3B	0.9700	C20—C25	1.391 (2)
C4—C5	1.526 (3)	C20—C21	1.402 (2)
C4—H4A	0.9700	C21—C22	1.382 (3)
C4—H4B	0.9700	C22—C23	1.387 (3)
C5—C6	1.533 (3)	C22—H22	0.9300
C5—H5A	0.9700	C23—C24	1.393 (2)
C5—H5B	0.9700	C23—H23	0.9300
C6—C7	1.514 (3)	C24—C25	1.396 (2)
C6—H6A	0.9700	C24—C26	1.537 (2)
C6—H6B	0.9700	C25—H25	0.9300
C7—H7	0.9800	C26—C29	1.531 (3)
C8—C9	1.514 (2)	C26—C27	1.532 (3)
C8—H8A	0.9700	C26—C28	1.534 (3)
C8—H8B	0.9700	C27—H27A	0.9600
C9—C14	1.387 (2)	C27—H27B	0.9600
C9—C10	1.404 (2)	C27—H27C	0.9600
C10—C11	1.383 (3)	C28—H28A	0.9600
C11—C12	1.387 (3)	C28—H28B	0.9600
C11—H11	0.9300	C28—H28C	0.9600
C12—C13	1.396 (2)	C29—H29A	0.9600
C12—H12	0.9300	C29—H29B	0.9600
C13—C14	1.395 (2)	C29—H29C	0.9600
			
C10—O1—H1O1	106.4	C13—C14—H14	118.4
C21—O2—H1O2	102.7	C18—C15—C16	108.66 (16)
C7—N1—C8	114.76 (13)	C18—C15—C13	111.78 (15)
C7—N1—C1	105.78 (13)	C16—C15—C13	108.71 (15)
C8—N1—C1	113.91 (13)	C18—C15—C17	108.22 (16)
C1—N2—C2	104.36 (12)	C16—C15—C17	108.85 (17)
C1—N2—C19	111.45 (12)	C13—C15—C17	110.56 (14)
C2—N2—C19	115.31 (12)	C15—C16—H16A	109.5
N2—C1—N1	106.28 (12)	C15—C16—H16B	109.5
N2—C1—H1A	110.5	H16A—C16—H16B	109.5
N1—C1—H1A	110.5	C15—C16—H16C	109.5
N2—C1—H1B	110.5	H16A—C16—H16C	109.5
N1—C1—H1B	110.5	H16B—C16—H16C	109.5
H1A—C1—H1B	108.7	C15—C17—H17A	109.5
N2—C2—C7	100.86 (12)	C15—C17—H17B	109.5
N2—C2—C3	119.27 (14)	H17A—C17—H17B	109.5
C7—C2—C3	110.48 (13)	C15—C17—H17C	109.5
N2—C2—H2	108.6	H17A—C17—H17C	109.5
C7—C2—H2	108.6	H17B—C17—H17C	109.5
C3—C2—H2	108.6	C15—C18—H18A	109.5
C2—C3—C4	107.44 (15)	C15—C18—H18B	109.5
C2—C3—H3A	110.2	H18A—C18—H18B	109.5
C4—C3—H3A	110.2	C15—C18—H18C	109.5
C2—C3—H3B	110.2	H18A—C18—H18C	109.5
C4—C3—H3B	110.2	H18B—C18—H18C	109.5
H3A—C3—H3B	108.5	N2—C19—C20	110.98 (13)
C5—C4—C3	112.87 (16)	N2—C19—H19A	109.4
C5—C4—H4A	109.0	C20—C19—H19A	109.4
C3—C4—H4A	109.0	N2—C19—H19B	109.4
C5—C4—H4B	109.0	C20—C19—H19B	109.4
C3—C4—H4B	109.0	H19A—C19—H19B	108.0
H4A—C4—H4B	107.8	C25—C20—C21	118.67 (16)
C4—C5—C6	112.16 (15)	C25—C20—C19	121.69 (15)
C4—C5—H5A	109.2	C21—C20—C19	119.64 (15)
C6—C5—H5A	109.2	O2—C21—C22	119.48 (15)
C4—C5—H5B	109.2	O2—C21—C20	120.80 (15)
C6—C5—H5B	109.2	C22—C21—C20	119.71 (16)
H5A—C5—H5B	107.9	C21—C22—C23	120.34 (16)
C7—C6—C5	108.19 (16)	C21—C22—H22	119.8
C7—C6—H6A	110.1	C23—C22—H22	119.8
C5—C6—H6A	110.1	C22—C23—C24	121.78 (16)
C7—C6—H6B	110.1	C22—C23—H23	119.1
C5—C6—H6B	110.1	C24—C23—H23	119.1
H6A—C6—H6B	108.4	C23—C24—C25	116.80 (15)
N1—C7—C2	101.63 (13)	C23—C24—C26	121.90 (15)
N1—C7—C6	115.78 (15)	C25—C24—C26	121.16 (15)
C2—C7—C6	111.66 (14)	C20—C25—C24	122.69 (16)
N1—C7—H7	109.1	C20—C25—H25	118.7
C2—C7—H7	109.1	C24—C25—H25	118.7
C6—C7—H7	109.1	C29—C26—C27	108.02 (15)
N1—C8—C9	111.07 (13)	C29—C26—C28	108.58 (16)
N1—C8—H8A	109.4	C27—C26—C28	108.83 (16)
C9—C8—H8A	109.4	C29—C26—C24	111.28 (14)
N1—C8—H8B	109.4	C27—C26—C24	111.77 (15)
C9—C8—H8B	109.4	C28—C26—C24	108.30 (14)
H8A—C8—H8B	108.0	C26—C27—H27A	109.5
C14—C9—C10	118.58 (15)	C26—C27—H27B	109.5
C14—C9—C8	121.12 (15)	H27A—C27—H27B	109.5
C10—C9—C8	120.25 (15)	C26—C27—H27C	109.5
O1—C10—C11	119.16 (15)	H27A—C27—H27C	109.5
O1—C10—C9	121.45 (15)	H27B—C27—H27C	109.5
C11—C10—C9	119.38 (15)	C26—C28—H28A	109.5
C10—C11—C12	120.79 (16)	C26—C28—H28B	109.5
C10—C11—H11	119.6	H28A—C28—H28B	109.5
C12—C11—H11	119.6	C26—C28—H28C	109.5
C11—C12—C13	121.37 (15)	H28A—C28—H28C	109.5
C11—C12—H12	119.3	H28B—C28—H28C	109.5
C13—C12—H12	119.3	C26—C29—H29A	109.5
C14—C13—C12	116.71 (15)	C26—C29—H29B	109.5
C14—C13—C15	120.97 (15)	H29A—C29—H29B	109.5
C12—C13—C15	122.28 (15)	C26—C29—H29C	109.5
C9—C14—C13	123.14 (16)	H29A—C29—H29C	109.5
C9—C14—H14	118.4	H29B—C29—H29C	109.5

### Hydrogen-bond geometry (Å, °)

**Table:**

*Cg*3 and *Cg*4 are the centroids of the C9–C14 and C20–C25 benzene rings, respectively.

**Table:**

*D*—H···*A*	*D*—H	H···*A*	*D*···*A*	*D*—H···*A*
O1—H1O1···N1	0.98	1.77	2.6585 (18)	148
O2—H1O2···N2	0.89	1.86	2.6794 (18)	153
C2—H2···O2^i^	0.98	2.45	3.367 (18)	155
C5—H5B···Cg3^ii^	0.96	2.87	3.625 (2)	135
C19—H19A···Cg4^iii^	0.96	2.84	3.6722 (18)	144

Symmetry codes: (i) *x*+1, *y*, *z*; (ii) −*x*+1, −*y*+1, −*z*+1; (iii) −*x*, −*y*+1, −*z*+2.
